# Worldwide impacts of atmospheric vapor pressure deficit on the interannual variability of terrestrial carbon sinks

**DOI:** 10.1093/nsr/nwab150

**Published:** 2021-08-20

**Authors:** Bin He, Chen Chen, Shangrong Lin, Wenping Yuan, Hans W Chen, Deliang Chen, Yafeng Zhang, Lanlan Guo, Xiang Zhao, Xuebang Liu, Shilong Piao, Ziqian Zhong, Rui Wang, Rui Tang

**Affiliations:** State Key Laboratory of Earth Surface Processes and Resource Ecology, College of Global Change and Earth System Science, Beijing Normal University, Beijing 100875, China; Department of Application Research, Twenty First Century Aerospace Technology Co., Ltd., Beijing 100723, China; School of Atmospheric Sciences, Southern Marine Science and Engineering Guangdong Laboratory (Zhuhai), Sun Yat-sen University, Zhuhai 519082, China; School of Atmospheric Sciences, Southern Marine Science and Engineering Guangdong Laboratory (Zhuhai), Sun Yat-sen University, Zhuhai 519082, China; Department of Physical Geography and Ecosystem Science, Lund University, Lund S-223 64, Sweden; Regional Climate Group, Department of Earth Sciences, University of Gothenburg, Gothenburg S-40530, Sweden; State Key Laboratory of Earth Surface Processes and Resource Ecology, College of Global Change and Earth System Science, Beijing Normal University, Beijing 100875, China; State Key Laboratory of Earth Surface Processes and Resource Ecology, College of Global Change and Earth System Science, Beijing Normal University, Beijing 100875, China; Academy of Disaster Reduction and Emergency Management, School of Geography, Beijing Normal University, Beijing 100875, China; State Key Laboratory of Remote Sensing Science, Faculty of Geographical Science, Beijing Normal University, Beijing 100875, China; State Key Laboratory of Earth Surface Processes and Resource Ecology, College of Global Change and Earth System Science, Beijing Normal University, Beijing 100875, China; Sino-French Institute for Earth System Science, College of Urban and Environmental Sciences, Peking University, Beijing 100871, China; State Key Laboratory of Earth Surface Processes and Resource Ecology, College of Global Change and Earth System Science, Beijing Normal University, Beijing 100875, China; State Key Laboratory of Earth Surface Processes and Resource Ecology, College of Global Change and Earth System Science, Beijing Normal University, Beijing 100875, China; State Key Laboratory of Earth Surface Processes and Resource Ecology, College of Global Change and Earth System Science, Beijing Normal University, Beijing 100875, China

**Keywords:** vapor pressure deficit, net ecosystem production, gross vegetation production, carbon dioxide concentration

## Abstract

Interannual variability of the terrestrial ecosystem carbon sink is substantially regulated by various environmental variables and highly dominates the interannual variation of atmospheric carbon dioxide (CO_2_) concentrations. Thus, it is necessary to determine dominating factors affecting the interannual variability of the carbon sink to improve our capability of predicting future terrestrial carbon sinks. Using global datasets derived from machine-learning methods and process-based ecosystem models, this study reveals that the interannual variability of the atmospheric vapor pressure deficit (VPD) was significantly negatively correlated with net ecosystem production (NEP) and substantially impacted the interannual variability of the atmospheric CO_2_ growth rate (CGR). Further analyses found widespread constraints of VPD interannual variability on terrestrial gross primary production (GPP), causing VPD to impact NEP and CGR. Partial correlation analysis confirms the persistent and widespread impacts of VPD on terrestrial carbon sinks compared to other environmental variables. Current Earth system models underestimate the interannual variability in VPD and its impacts on GPP and NEP. Our results highlight the importance of VPD for terrestrial carbon sinks in assessing ecosystems’ responses to future climate conditions.

## Teaser

Atmospheric vapor pressure deficit has worldwide impacts on the interannual variability of terrestrial carbon sinks and atmospheric carbon dioxide concentrations.

## INTRODUCTION

Atmospheric carbon dioxide (CO_2_) has substantially increased during the last century, and the concentration reached almost 410 ppm in 2019 [1]. Terrestrial ecosystems, as a major carbon sink, play an important role in regulating the global carbon cycle and atmospheric CO_2_ concentrations [2,3]. On average, terrestrial ecosystems absorbed atmospheric CO_2_ at a rate of 2.35 Pg C yr^–1^ during 1959–2019, a value that was 0.60 Pg C yr^–1^ larger than that of the ocean, another important carbon sink for the atmosphere [1]. The increased rate of terrestrial carbon sink (0.0415 Pg C yr^–1^) was significantly higher than that of the ocean carbon sink (0.0299 Pg C yr^–1^) [1] (Fig. S1a). In particular, the terrestrial carbon sink showed substantially larger interannual variability than did the ocean carbon sink (Fig. S1b), and the coefficient of variation of the land sink was 3.94 times that of the ocean sink. In addition, the land sink substantially regulated the year-to-year variations in the atmospheric CO_2_ growth rate (Fig. S2). Thus, understanding interannual variability and environmental regulation of the land sink is required for reducing large uncertainties in projections of the terrestrial carbon cycle and monitoring global atmospheric CO_2_ concentrations on a year-to-year basis.

Atmospheric vapor pressure deficit (VPD) has been identified as an increasingly important driver of plant functioning in terrestrial biomes and has been established as a major contributor to recent drought-induced plant mortality independent of other drivers associated with climate change. Physically it is a measure of how far the atmospheric water vapor is away from the maximum under a given temperature. Specifically, a high VPD would induce the closure of plant stomata to prevent extensive water loss [4–6], which subsequently suppresses the photosynthesis rate and decreases productivity [5,7]. In addition, a recent study suggested the emergence of VPD regulation on the tropical carbon cycle [8]. Nevertheless, the global constraints of VPD changes on terrestrial carbon sinks and atmospheric CO_2_ concentrations have not yet been quantified.

This study first examines the relationship between VPD and global terrestrial net ecosystem productivity (NEP) derived from machine-learning methods in the FLUXCOM product [9] and ecosystem process-based models from the TRENDY project [10,11]. Because these two datasets are independent of each other, the VPD-NEP relationships derived from them provide valuable independent evidence for the relationship. After removing the seasonal and long-term trends of NEP and the corresponding climatic variables, the interannual variation (IAV) in global NEP from both of these datasets exhibited high consistency with the variation in VPD at the global scale, implying VPD strongly regulates global terrestrial ecosystem carbon uptake and the atmospheric CO_2_ growth rate (CGR).

## RESULTS

**Figure 1.**
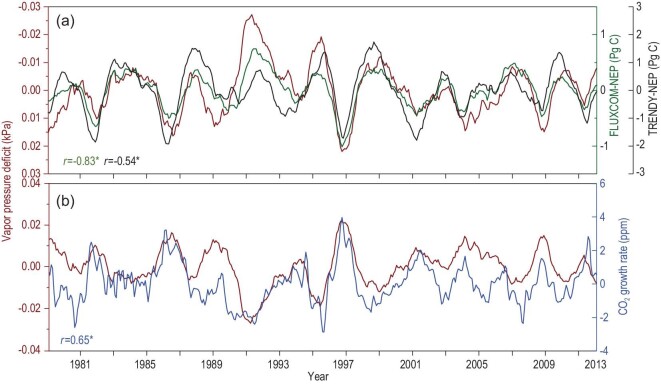
Correlations between the interannual variation in land carbon sinks (NEP), atmospheric CO_2_ growth rate and vapor pressure deficit (VPD) at the monthly scale. (a) Interannual variations in NEP and VPD over global lands from 1980 to 2013; NEP simulations from FLUXCOM (green line) and TRENDY (black line) were used. (b) Interannual variation in the atmospheric CO_2_ growth rate (blue line) and the VPD over global lands from 1980 to 2013. The numbers in the figure show the correlation coefficients (*r*) of the VPD with FLUXCOM-NEP (green), TRENDY-NEP (black) and CO_2_ growth rate (blue), and ^*^ indicates statistical significance at *P* < 0.05.

This study used two global model datasets (i.e. FLUXCOM and TRENDY, see Methods) to analyze the impacts of VPD on the interannual variability of terrestrial carbon sinks. The detrended monthly global VPD over land showed a significant negative correlation with the detrended monthly global terrestrial NEP over land derived from both the FLUXCOM and the TRENDY V8 datasets (Fig. 1a). The interannual variability in the CGR was highly consistent with the simulated NEP derived from the two datasets (Fig. S3), confirming the strong regulation of terrestrial carbon uptake on atmospheric CO_2_ concentrations. Therefore, the interannual variability in the measured atmospheric CGR also showed a significant negative correlation with the VPD (*P* < 0.05) (Fig. 1b), which implies that the VPD had strong regulatory effects on the interannual variability of the CGR. At the yearly scale, the correlations between VPD and NEP/CGR were also robust, as shown in Fig. S4.

**Figure 2.**
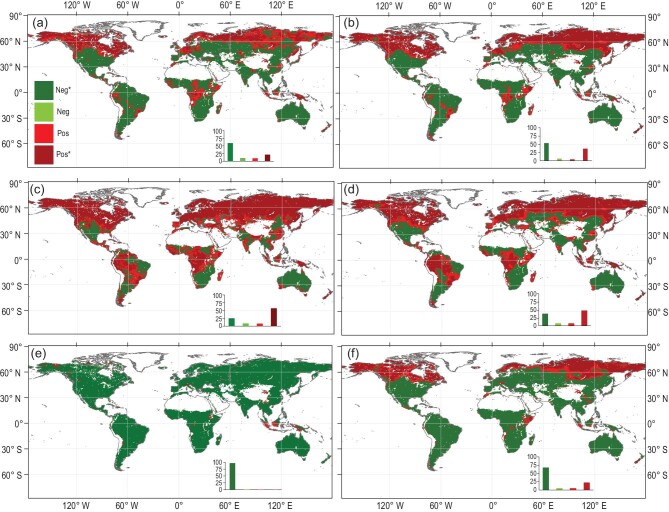
Spatial patterns of (a and b) correlations of VPD with gross primary production, (c and d) ecosystem respiration, and (e and f) net ecosystem production derived from FLUXCOM and TRENDY. The left column indicates FLUXCOM and the right column indicates TRENDY. The insets show the relative frequency (%) distribution of significant negative correlations (Neg^*^; *P* < 0.05; dark green), negative correlations (Neg; light green), positive correlations (Pos; light red) and significant positive correlations (Pos^*^; *P* < 0.05; dark red).

We further investigated the impacts of VPD on NEP in terms of global patterns. In 98.8% of vegetated areas, detrended monthly NEP simulations derived from FLUXCOM showed a negative correlation with detrended VPD (∼97.5% with a significant negative correlation) (Fig. 2e). Similarly, detrended TRENDY NEP negatively correlated with VPD over 72.8% of the areas (∼67.8% with significant correlation) (Fig. 2f). The response of NEP to VPD in each ecosystem type was also examined, as shown in Fig. S5. Over almost all ecosystem types, NEP derived from TRENDY and FLUXCOM showed negative correlations with interannual variability of VPD (Fig. S5). Both NEPs from TRENDY and FLUXCOM indicated that evergreen broadleaf forests are most sensitive to VPD change. TREND and FLUXCOM disagree about the relationship between NEP and VPD in deciduous needleleaf forests, where TRENDY showed a positive correlation between NEP and VPD, whereas FLUXCOM suggested a negative relationship. This difference is also evident in the spatial distribution of NEP-VPD correlations (Fig. 2e and f), implying uncertainties exist among different data sources.

Ecosystem NEP is jointly determined by vegetation gross primary production (GPP) and terrestrial ecosystem respiration (TER), and their relationship can be written as NEP = GPP−TER. Therefore, we analyzed the impacts of VPD on GPP and TER. The simulated GPP derived from the FLUXCOM and TRENDY datasets showed relatively consistent correlations with the VPD in the low- and mid-latitudes (Fig. 2a and b), and a higher VPD constrained the vegetation GPP. In northern high latitudes, the GPP derived from the TRENDY dataset revealed a positive sensitivity to VPD variation.

**Figure 3.**
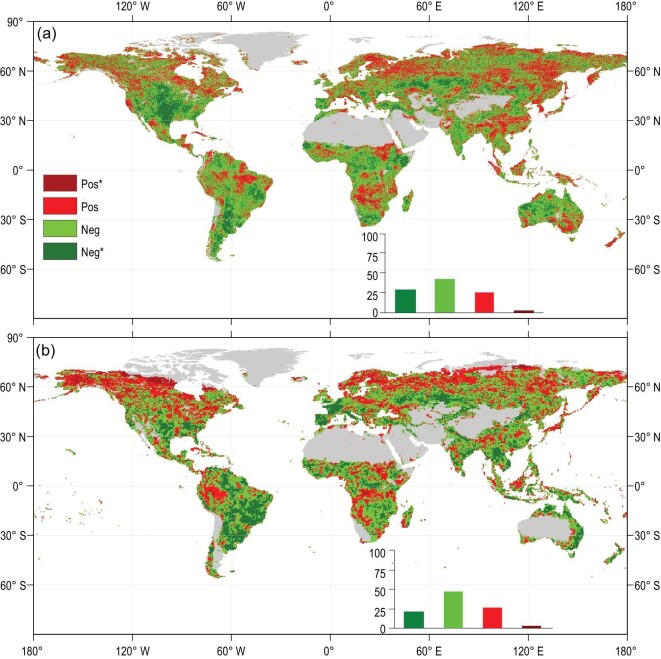
Spatial patterns of correlations between VPD and satellite-based (a) NIRv and (b) CSIF. The insets show the relative frequency (%) distribution of significant negative correlations (Neg^*^; *P* < 0.05; dark green), negative correlations (Neg; light green), positive correlations (Pos; light red) and significant positive correlations (Pos^*^; *P* < 0.05; dark red).

In addition, we used a satellite-based vegetation index (NIRv, near-infrared reflectance of vegetation) and contiguous solar-induced fluorescence (CSIF) measurements to reveal the large‐scale coupling between VPD and vegetation growth. The analyses based on two satellite-based datasets, the NIRv and the CSIF datasets, confirm the dominant roles of VPD in regulating global vegetation growth. From 1982 to 2015, ∼69.3% of the vegetation surface showed a negative correlation of interannual variability between NIRv and VPD (21.70% with a significant correlation; Fig. 3a). Similarly, from 2000 to 2015, the CSIF dataset showed that the interannual variability of the VPD was negatively correlated with that of the CSIF over 71.40% of vegetated areas (28.50% with a significant correlation; Fig. 3b).

**Figure 4.**
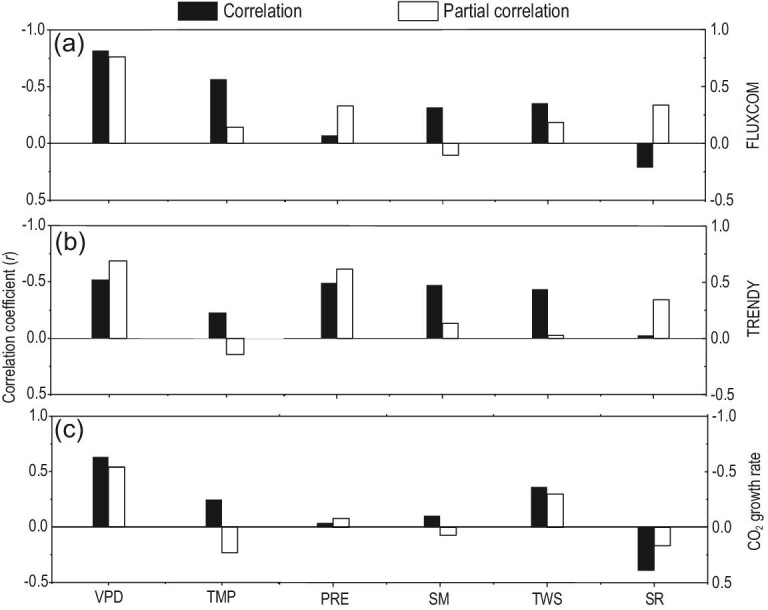
Correlation of environmental variables with (a and b) global mean net ecosystem production and (c) atmospheric CO_2_ growth rate. Net ecosystem production is derived from (a) FLUXCOM and (b) TRENDY. The correlation analysis includes black bars indicating the correlation coefficient and white bars indicating the partial correlation excluding other variables. VPD, vapor pressure deficit; TMP, air temperature; PRE, precipitation; SM, soil moisture; TWS, terrestrial water storage; SR, downwelling shortwave radiation.

As highlighted in previous studies [12–19], temperature and land water storage substantially regulated the interannual variability of terrestrial carbon sinks and the CGR. We further compared the impacts of VPD, temperature, precipitation, soil moisture, land total water storage and downwelling shortwave radiation on NEP. The magnitudes of the impacts were estimated using partial correlation analysis to exclude the impacts of other variables when investigating the impact of a given variable. This analysis reveals that the global VPD-NEP relationship remained significant after controlling for the effects of air temperature, precipitation, soil moisture, land water storage and radiation (partial correlations of −0.76 and −0.69, respectively; Fig. 4). The analysis also indicates significant correlations of detrended VPD with detrended CGR after excluding the impacts of other environmental variables (Fig. 4c). In contrast, controlling for the effect of VPD strongly decreased the partial correlations of NEP and CGR with other environmental variables (i.e. air temperature, precipitation, land water storage and radiation) (Fig. 4). Spatially, we also observed a widespread negative correlation between NEP and VPD, especially for NEP from FLUXCOM (Figs S6 and S7). Simultaneously, relatively high positive correlations between NEP and precipitation were also found in a large area. Our results highlight that VPD had stronger effects on the interannual variability of NEP and CGR than did other environmental variables.

**Figure 5.**
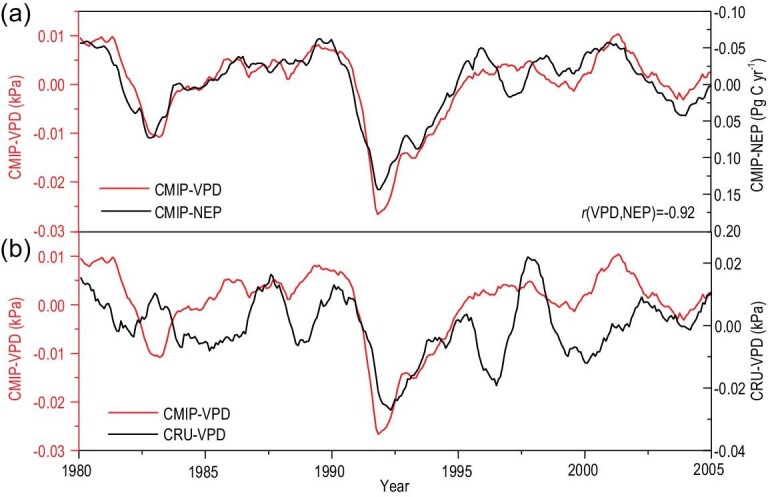
Interannual variations in the NEP and VPD simulated by CMIP5. The numbers in the figure show the correlation coefficients (*r*).

Having established the significant relationship between VPD and NEP on the interannual scale by empirical and ecosystem model data, it would be interesting to examine whether the current Earth system models (ESMs) could accurately reproduce the interannual variability of VPD and its impact on terrestrial carbon sink. Using simulations from 19 CMIP5 (Coupled Model Intercomparison Project Phase 5) ESMs, we find that the interannual variability of the VPD simulated by the CMIP5 models shows a strong correlation with the terrestrial NEP (Fig. 5a). However, although the models can capture the decadal trend of the observed VPD, they give a poor performance with regard to reproducing the absolute magnitude and interannual variability of the VPD in the CRU dataset (Fig. 5b). Therefore, although the current ESMs can fairly well reproduce the relationship between VPD and the terrestrial NEP, the poor performance when reproducing the interannual variability of VPD substantially limits their capability to reproduce the interannual variability of the NEP.

## DISCUSSION

Our results highlight the dominant role of the VPD in the interannual variability of terrestrial ecosystem carbon fluxes (i.e. GPP and NEP) as well as atmospheric CGR. Other lines of evidence support our conclusion that VPD has profound negative impacts on plant productivity [20–23]. For example, Novick *et al.* [20] decoupled the impact of the soil moisture supply and atmospheric water demand (indicated by the VPD) on plant stomatal conductance and suggested a greater constraint of the latter than the former on stomatal conductance for many biomes, and thus on the ecosystem water and carbon fluxes. Grossiord *et al.* [23] also suggested that, when the VPD exceeds a certain threshold, plant photosynthesis and growth for most species will be limited, resulting in a higher risk for hydraulic failure and carbon starvation. Recently, Yuan *et al.* [2] observed an apparent shift in global vegetation greenness from greening to browning in the 1990s due to a sharp increase in VPD. Although the VPD has few direct influences on TER change, the high VPD associated with high temperature and low soil moisture would have a great impact on TER. We observed a contrasting pattern of the VPD-TER relationship between arid and humid ecosystems (Fig. 2c and d). In humid regions, there are positive relationships between VPD and TER that mostly result from associated increases of air temperature with VPD. In contrast, over the semi-arid and arid ecosystems, water availability is the most important dominating variable for TER, and thus water stress accompanying high VPD may limit TER [12,24,25]. A great regulation of VPD on the tropical land carbon cycle was discovered by a previous study [8], implying a dominant role of the tropical forest in the established relationship between VPD and NEP on the global scale. This study provides profound and direct evidence for the impacts of VPD on global NEP and the global atmospheric CGR.

Several climate variables have been found to substantially regulate the interannual variability of carbon uptake by terrestrial ecosystems globally [12–19]. The strong sensitivity of terrestrial carbon fluxes to temperature [13,14] and land water storage [18] has been documented. However, whether the interannual variability of the terrestrial carbon sink responds to temperature or water storage is still controversial [12]. An important reason for the current debate is likely that the impact of the VPD on terrestrial carbon sinks has been ignored. It is known that soil moisture and temperature are closely linked to VPD [26,27]. Therefore, a link between VPD and temperature and soil moisture can be expected. Inevitably, the impact of VPD on the IAV of NEP is directly or indirectly dependent upon temperature and soil moisture conditions [19]. This study highlights the strong and worldwide impacts of VPD on the interannual variability of terrestrial carbon sinks, which should be adequately considered in order to quantify the role of climate change in the global carbon cycle.

## DATA AND METHODS

### Terrestrial carbon cycle datasets

Three global land carbon flux datasets, FLUXCOM [9], TRENDY [10] and CMIP5, were used to explore the interannual variation in global land vegetation GPP, TER and NEP. The FLUXCOM product was produced by machine-learning algorithms based on training using *in situ* flux observations for the period of 1980 to 2013. The global GPP, TER and NEP were estimated at the monthly scale with a spatial resolution of 0.5° × 0.5°. TRENDY is an ensemble of simulations of dynamic global vegetation models (DGVMs) from 1900 to the present [10], forced by observed climate data. In this study, the multi-model ensemble mean of the simulated GPP, TER and NEP from 12 DGVMs (Table S1) in TRENDY v8 were used. A total of 17 DGVMs joined the TRENDY project, but 5 DGVMs were excluded here due to missing NEP output.

Historical simulations of CMIP5 models were derived from 19 ESMs in this study (Table S2). The estimates of land and ocean carbon sink data from 1959 to 2019 used in this study were provided by the Global Carbon Budget 2020 (https://doi.org/10.18160/gcp-2020).

### Atmospheric CO_2_ concentration data

The monthly atmospheric CO_2_ concentration time series from 1959 was obtained from the Greenhouse Gas Marine Boundary Layer Reference (MBL) of the National Oceanic and Atmospheric Administration Earth System Research Laboratory (NOAA/ESRL). The time series of CO_2_ concentrations from 1959 to 1980 was compiled based on records of the Mauna Loa and South Pole stations, while data from 1980 to the present were compiled from records of multiple NOAA/ESRL stations.

The monthly atmospheric CO_2_ concentration records were obtained from the GLOBALVIEW-CO_2_ product, which provides observations of atmospheric CO_2_ concentration over 313 global air-sampling sites with 7-day intervals (NOAA Global Monitoring Division, 2013). If the ratio of missing data is >20% for a given year, then this year was indicated as ‘missing’. The sites with at least 10 years of observations were included in this study to calculate global mean CO_2_ concentration. Eventually, 77 sites were included equally in the calculation of global monthly mean CO_2_ concentration without any weighting of individual sites.

### Climate and satellite datasets

Global air temperature, vapor pressure and precipitation data derived from the Climate Research Unit (CRU v4.02) [28] were used to analyze the impacts of the interannual variability of climate variables on the terrestrial carbon cycle. CRU provides monthly climate variables with a spatial resolution of 0.5° × 0.5° from 1900 to the present. The monthly downwelling shortwave radiation data with a spatial resolution of 0.5° × 0.625° from 1980 to the present were collected from the MERRA2 re-analysis product [29]. In addition, this study included historical simulations of climate variables from 18 models of CMIP5 (Table S1).

The VPD was calculated based on CRU and CMIP5 climate datasets. The method [30,31] for calculating the VPD was as follows:

(1) \begin{equation*} {\rm{VPD\ }} = {\rm{\ SVP}} - {\rm{AVP}}, \end{equation*}

(2) \begin{eqnarray*} {\rm{SVP}} = 0.5 \times \left( 0.611 \times \exp \left( \frac{17.3 \times {T_{\rm min}}}{{{T_{\rm min}} + 237.3}} \right) \right.\nonumber\\ \left. +\, 0.611 \times \exp \left( {\frac{{17.3 \times {T_{\rm max}}}}{{{T_{\rm max}} + 237.3}}} \right) \right), \end{eqnarray*}

where SVP and AVP are the saturated vapor pressure and actual vapor pressure (kPa), respectively. *T*_max_ and *T*_min_ are the maximum air temperature and minimum air temperature (°C), respectively.

Equation (2) should ideally be calculated using daily maximum and minimum temperatures, but the CRU dataset provides only monthly averages of these variables. This is however not a problem because the relationship between temperature and saturated water vapor pressure is close to linear for a small range of temperature fluctuations. A comparison of monthly VPD anomalies calculated using daily and monthly data from ERA5 (https://www.ecmwf.int/en/forecasts/datasets/reanalysis-datasets/era5) confirms that using daily and monthly data produces similar results, see Figs S8 and S9.

Global terrestrial water storage (TWS) data for the period 1980–2016 were provided by Humphrey *et al.* [32]. This TWS series was reconstructed based on TWS measurements derived from the Gravity Recovery and Climate Experiment (GRACE) relying on a statistical model, which was forced by daily precipitation and temperature data [32].

The daily soil moisture with a spatial resolution of 0.25° for the period 1980–2018 was obtained from the Global Land Evaporation Amsterdam Model (GLEAM v3.3a) [33].

Global NIRv [34] data from 1982 to 2015 were derived from the Advanced Very High Resolution Radiometer (AVHRR) with red (*ρ_r_*) and near-infrared (*ρ_nir_*) band reflectance:

(3) \begin{equation*} {\rm{NIRv}} = \frac{{{\rho _{nir}} - {\rho _r}}}{{{\rho _{nir}} + {\rho _r}}}\ \times {\rho _{nir}}\!. \end{equation*}

In each year, the yearly NIRv data were the mean value of monthly cloud-free NIRv during the growing season (defined as the period when the monthly mean air temperature is higher than 0°C).

Global solar-induced fluorescence (SIF) data were obtained from a contiguous solar-induced fluorescence (CSIF) dataset [35]. The yearly SIF data were the average number from all the CSIF all-daily data during the growing season.

The global land cover map was derived from the MODIS land cover product (MCD12C1) with a spatial resolution of 0.05° (https://modis.gsfc.nasa.gov/data/dataprod/mod12.php), and the map of 2012 was used for the analysis. Global continents were divided into eight main types of ecosystems (Fig. S10) according to LAI/fpar classification scheme.

### Analysis of interannual variability

In line with previous studies, the monthly atmospheric CGR was defined as the difference in the CO_2_ concentrations between two successive months. Then, the seasonal cycle and long-term trends were removed by subtracting the historical mean value and applying a simple linear regression according to Refs [13,18]. Finally, a 12-month running sum was used to convert the month value to annual CGR. Similarly, the seasonal cycle and long-term trends of environmental and carbon variables (GPP, TER, NEP) were excluded, and the annual values were obtained. Note that a 12-month running average rather than the sum of 12 months was used to obtain the annual value of environmental variables, except precipitation.

## Supplementary Material

nwab150_Supplemental_File
