# Supplementary material for: Worldwide impacts of atmospheric vapor pressure deficit on the interannual variability of terrestrial carbon sinks
Source: Natl Sci Rev. 2021 Aug 20;9(4):nwab150. doi: 10.1093/nsr/nwab150 (PMC8982191; doi:10.1093/nsr/nwab150)
Supplement: nwab150_Supplemental_File [file nwab150_supplemental_file.pdf]

SUPPORTING INFORMATION OF

Worldwide impacts of atmospheric vapor pressure deficit on the interannual variability of terrestrial carbon sinks

Bin He[1*], Chen Chen[2], Shangrong Lin[3], Wenping Yuan[3*], Hans W. Chen[4], Deliang Chen[5], Yafeng Zhang[1], Lanlan Guo[1, 6], Xiang Zhao[7], Xuebang Liu[1], Shilong Piao[8], Ziqian Zhong[1], Rui Wang[1], Rui Tang[1]

[1] State Key Laboratory of Earth Surface Processes and Resource Ecology, College of Global Change and Earth System Science, Beijing Normal University, Beijing 100875, China

[2] Twenty First Century Aerospace Technology Co., Ltd., Beijing 100723, China

[3] School of Atmospheric Sciences, Southern Marine Science and Engineering Guangdong Laboratory (Zhuhai), Sun Yat-sen University, Zhuhai 519082, China

[4] Department of Physical Geography and Ecosystem Science, Lund University, Lund S-223 64, Sweden

[5] Regional Climate Group, Department of Earth Sciences, University of Gothenburg, Gothenburg S-40530, Sweden

[6] Academy of Disaster Reduction and Emergency Management, School of Geography, Beijing Normal University, Beijing 100875, China

[7] State Key Laboratory of Remote Sensing Science, Faculty of Geographical Science, Beijing Normal University, Beijing 100875, China

[8] Sino-French Institute for Earth System Science, College of Urban and Environmental Sciences, Peking University, Beijing 100871, China

[*] Corresponding authors. E-mails: hebin@bnu.edu.cn; yuanwp3@mail.sysu.edu.cn

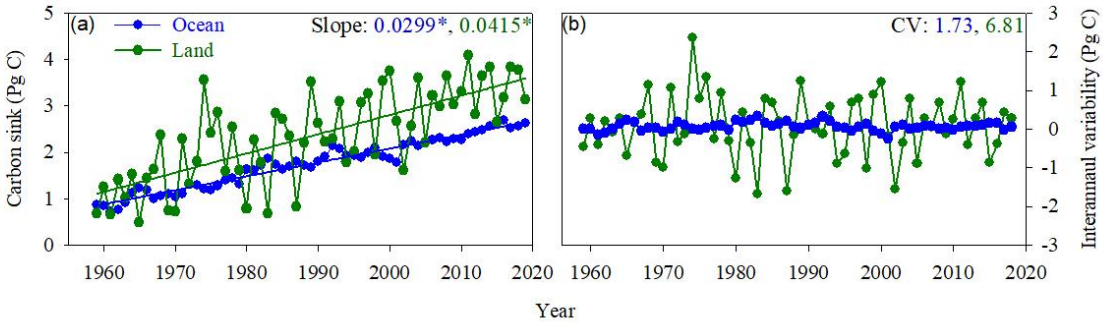

Fig. S1. Long-term trend and variability of land and ocean carbon sink. (a) Long-term trend of land and ocean carbon sinks. The numbers indicate the slopes of carbon sink with year (blue for ocean, green for land), and * indicate the significant trend (*p*<0.05). (b) Detrended interannual variability of land and ocean carbon sink. The numbers indicate the coefficient of variance of carbon sink (blue for ocean, green for land).

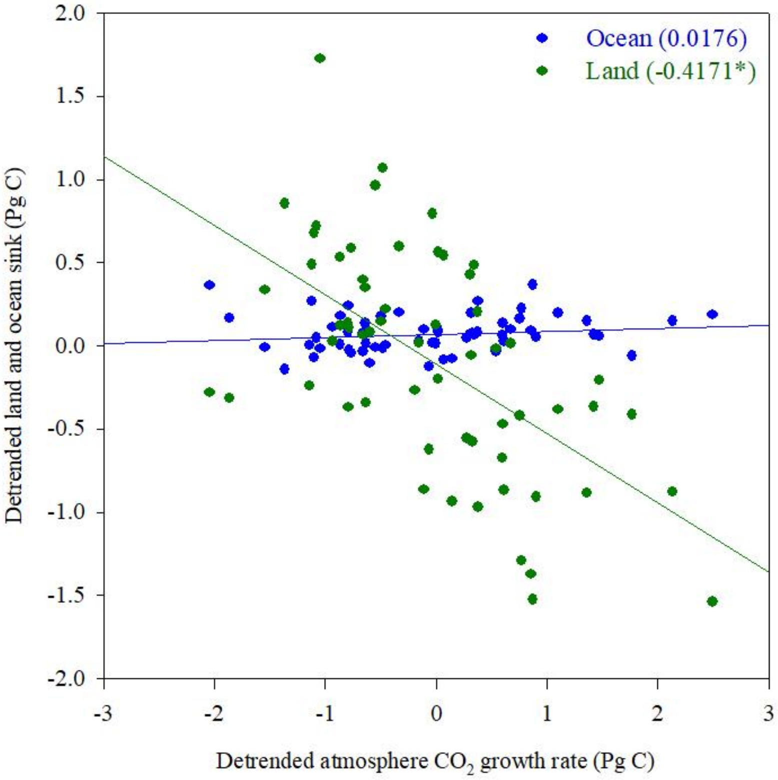

Fig. S2. Correlation of detrended annual atmospheric CO_2 growth rate with detrended land and ocean carbon sinks. Land and ocean carbon sink are derived from Friedlingstein et al. (2020). The numbers in the figure show the correlation coefficients (*r*), and * indicate the statistical significance at $p < 0.05$.

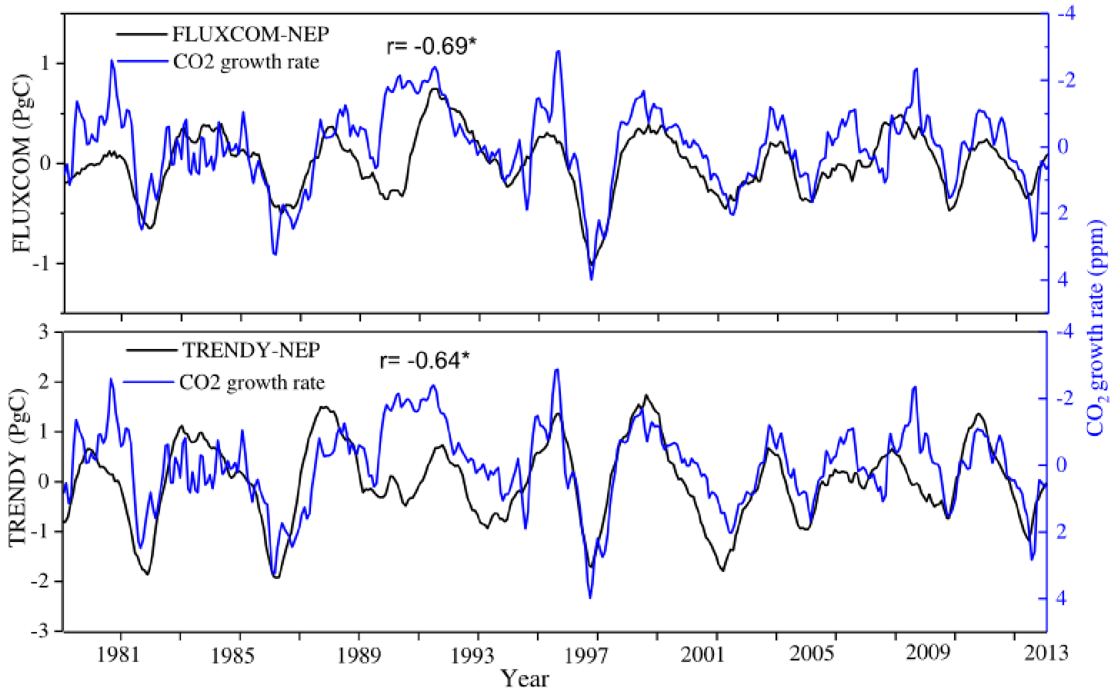

Fig. S3. Correlations between interannual variation of land carbon sink (NEP) and atmospheric CO_2 growth rate. NEP simulations from FLUXCOM (a) and TRENDY (b) were used. The numbers in the figure show the correlation coefficients (*r*), and * indicate the statistical significance at $p < 0.05$.

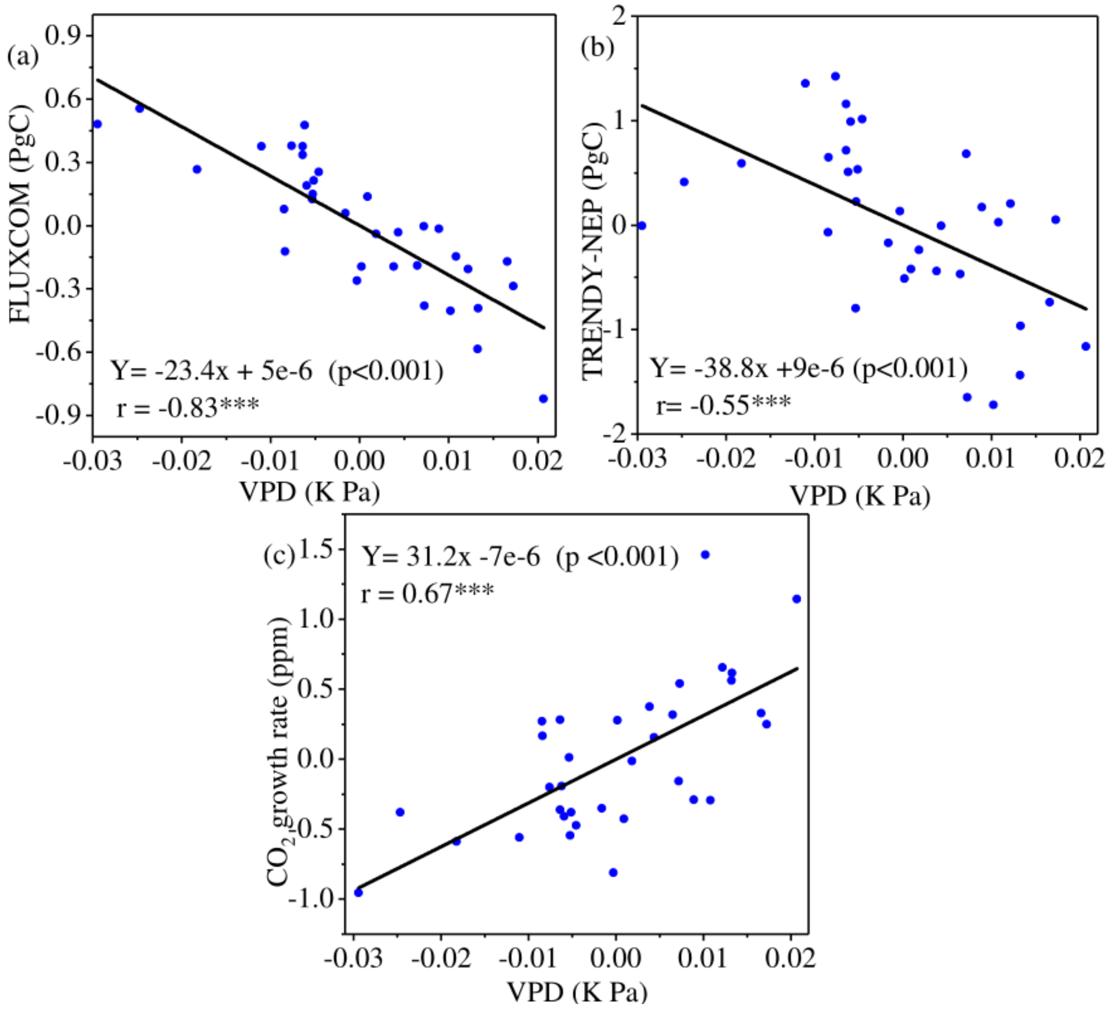

Fig. S4. The scatter plots of yearly VPD and NEP and atmospheric CO_2 growth rate. NEP simulations from FLUXCOM (a) and TRENDY (b) were used. *** indicates statistical significance at $p < 0.001$.

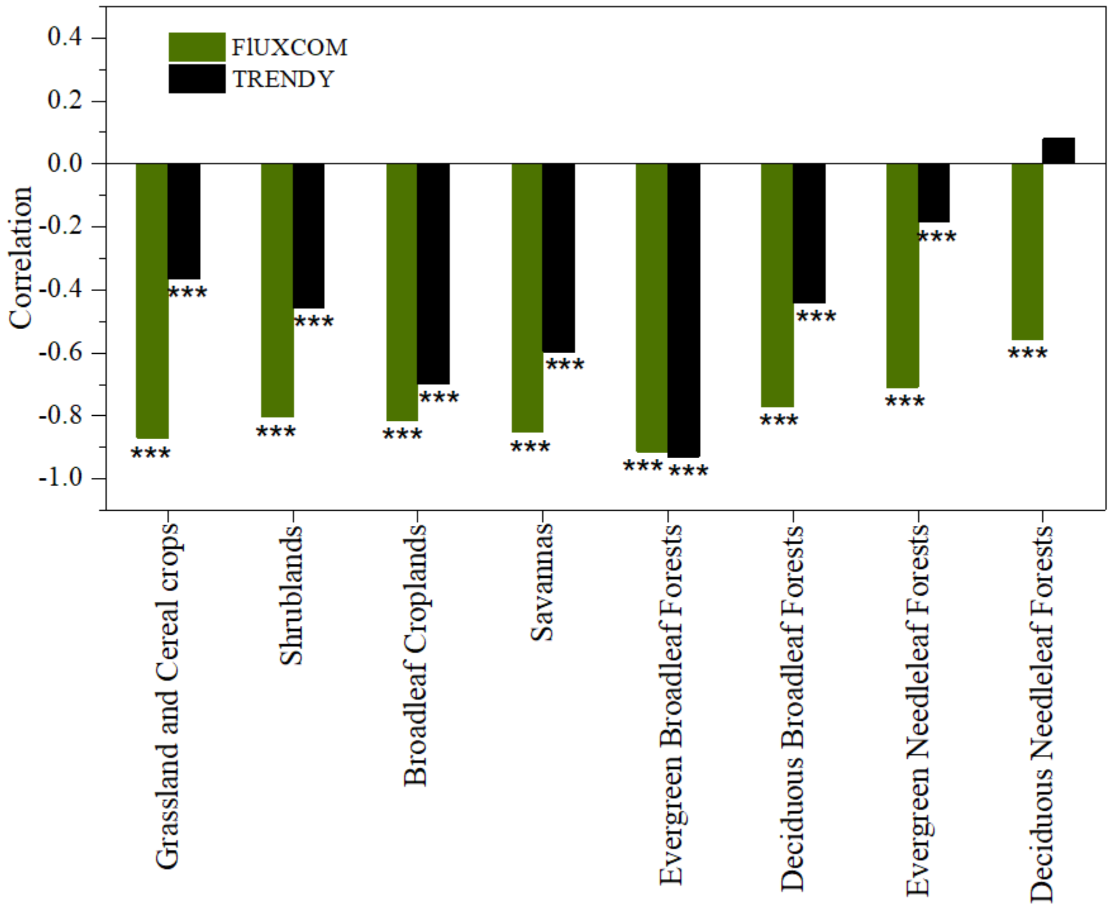

Fig. S5. The correlation between NEP and VPD at biomes scales. The green columns indicate NEP from FLUXCOM, and the black ones are from TRENDY. *** indicates statistical significance at $p < 0.001$.

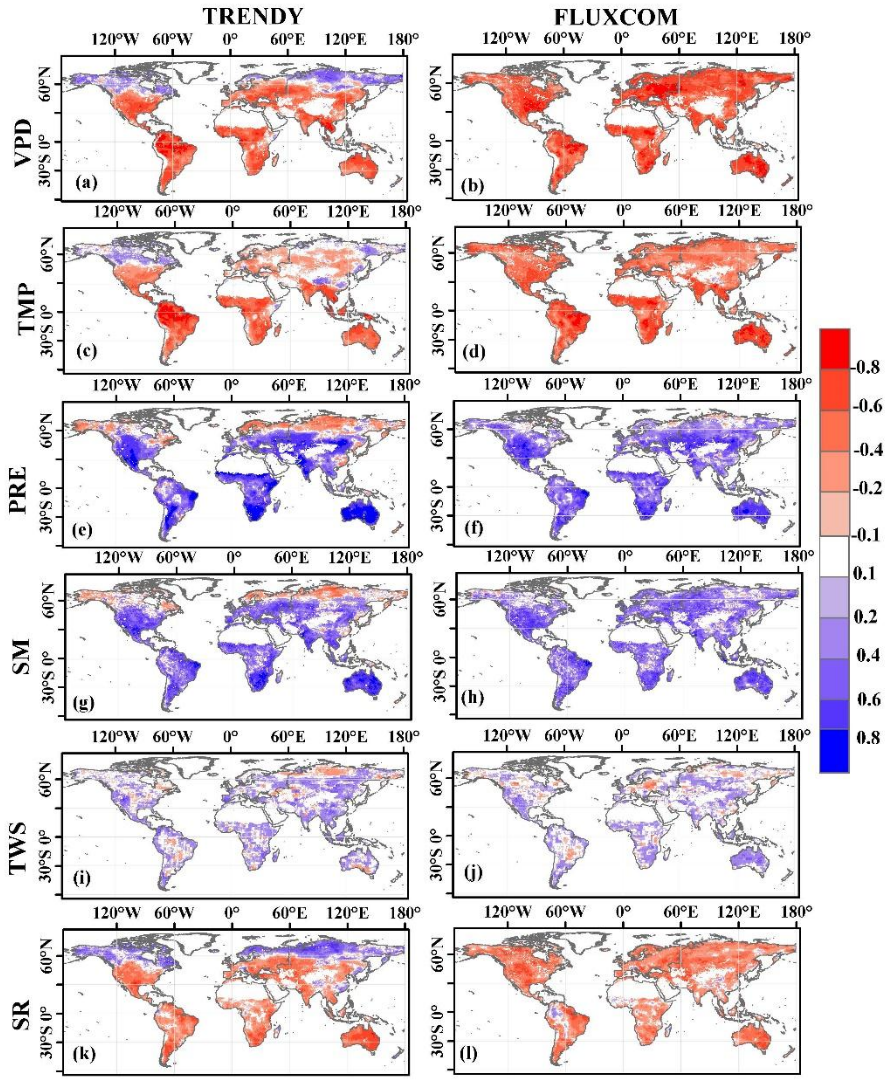

Fig. S6. Spatial pattern of correlations between NEP and environmental variables. Net ecosystem production is derived from TRENDY (a-i) and FLUXCOM (b-j). VPD: vapor pressure deficit; TMP: air temperature; PRE: precipitation; TWS: terrestrial water storage; SR: downwelling shortwave radiation.

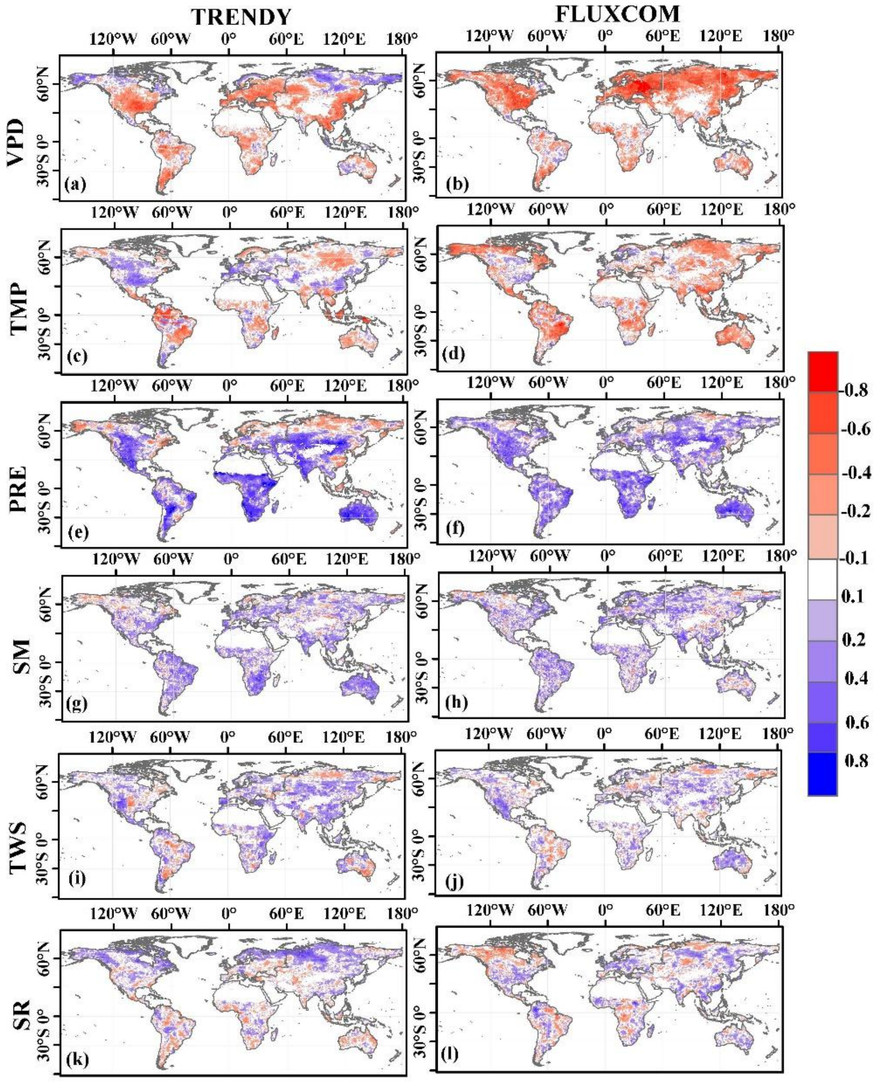

Fig. S7. Spatial pattern of partial correlations between NEP and environmental variables. Net ecosystem production is derived from TRENDY (a-i) and FLUXCOM (b-j). VPD: vapor pressure deficit; TMP: air temperature; PRE: precipitation; TWS: terrestrial water storage; SR: downwelling shortwave radiation.

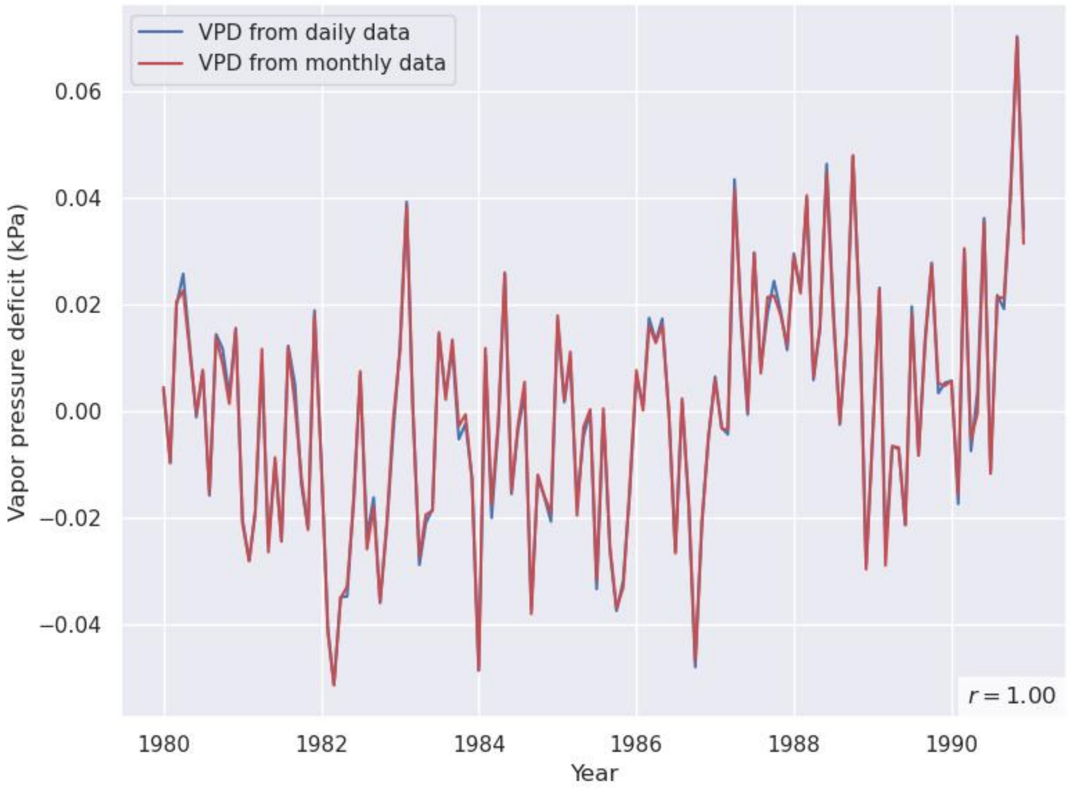

Fig. S8. Monthly anomalies of global averaged VPD calculated using daily and monthly climate data (1980-1990), respectively. The climate data was obtained from the ERA5 reanalysis. Only grid cells that are covered by at least 10% vegetation are included.

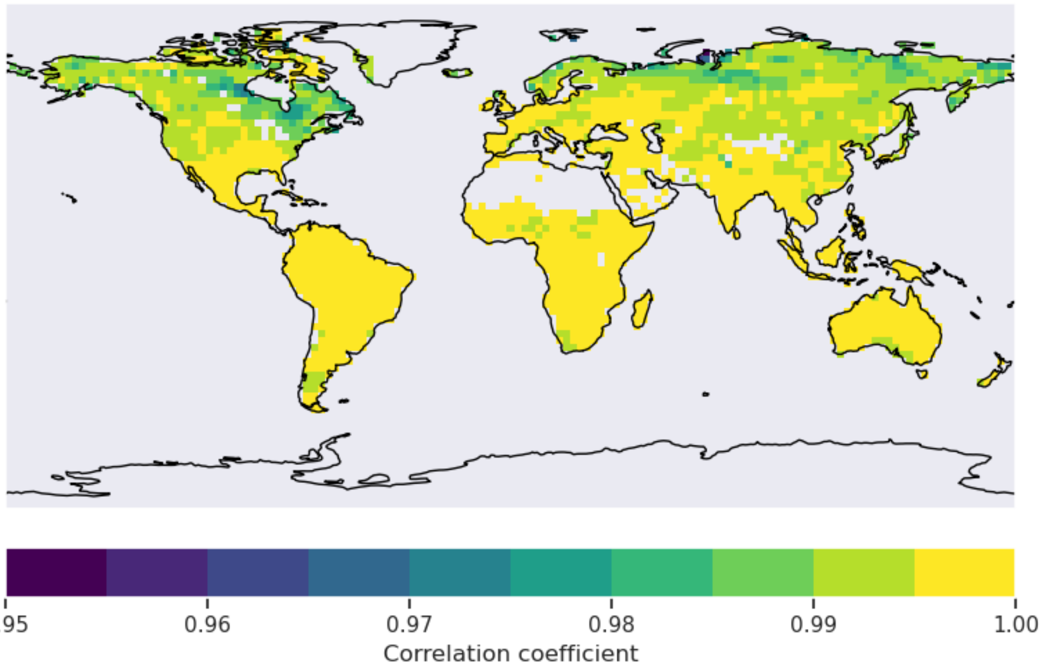

Fig. S9. Correlations between monthly anomalies of VPD calculated using daily and monthly climate data (1980-1990). The climate data was obtained from the ERA5 reanalysis. Only grid cells that are covered by at least 10% vegetation are included.

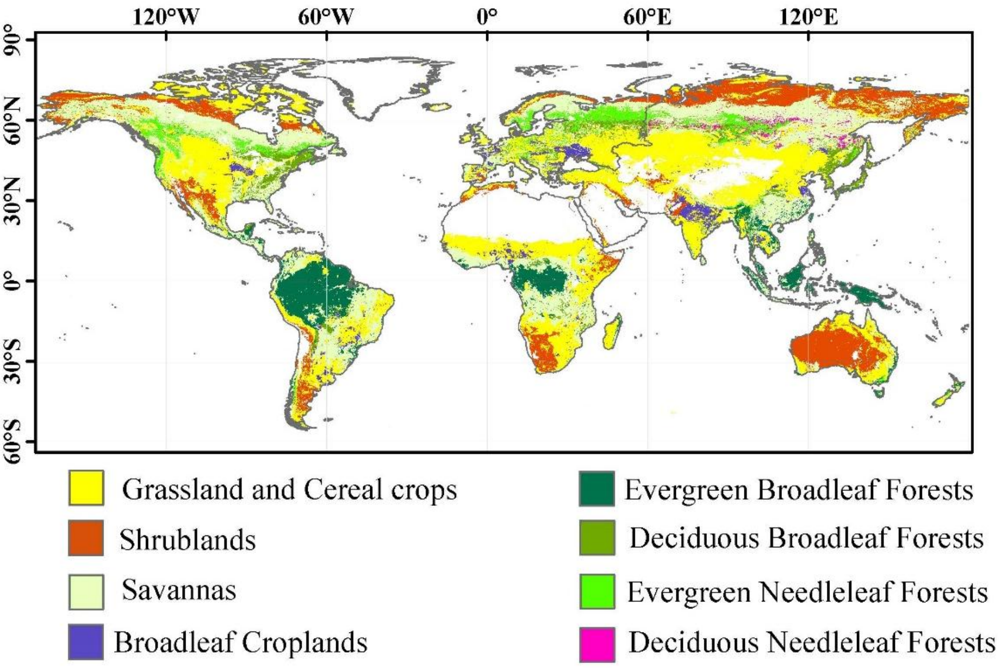

Fig.S10. Classification of global land cover. Global lands were divided into six main types based on MODIS land cover map (2012) according to Ahlström et al.'s (1) study.

Table S1. Information about nine terrestrial ecosystem models in TRENDY project used in this study.

Model name	Spatial resolution	Periods	Reference
CABLE	0.5 °×0.5 °	1860-2018	(2)
CLASS	2.8125 ° × 2.8125 °	1700-2018	(3)
CLM5.0	0.9375 ° × 1.25 °	1700-2018	(4)
DLEM	0.5 ° × 0.5 °	1700-2018	(5)
ISAM	0.5 ° × 0.5 °	1700-2018	(6)
ISBA-CYRIP	1 ° × 1 °	1700-2018	(7)
JSBACH	1.875 ° × 1.875 °	1700-2018	(8)
JULES	1.25 ° × 1.875 °	1700-2018	(9)
LPX	0.5 ° × 0.5 °	1700-2018	(10)
ORCHIDEE	0.5 ° × 0.5 °	1700-2018	(11)
SDGVM	0.5 ° × 0.5 °	1700-2018	(12)
VISIT	0.5 ° × 0.5 °	1860-2018	(13)

Table S2. Information about 19 CMIP5 Earth System Models used in this study.

**Institute**	**Modeling Center**	**Model Name**	**Resolution**	**Reference**
BCC	Beijing Climate Center, China Meteorological Administration	BCC-CSM1-1	2.8125 °×2.8125 °	(14)
		BCC-CSM1-1-M	1.125 °×1.125 °	
BNU	Beijing Normal University	BNU-ESM	2.8125 °×2.8125 °	(15)
CCCma	Canadian Centre for Climate Modeling and Analysis	CanESM2	2.8125 °×2.8125 °	(16)
NCAR	The National Center for Atmospheric Research	CCSM4	0.9 °×1.25 °	(17)
NOAA-GFDL	NOAA Geophysical Fluid Dynamics Laboratory	GFDL-ESM2G	2 °×2.5 °	(18, 19)
		GFDL-ESM2M	2 °×2.5 °	
NASA-GISS	NASA Goddard Institute for Space Studies	GISS-E2-H	2 °×2.5 °	(20)
		GISS-E2-H-CC	2 °×2.5 °	
		GISS-E2-R	2 °×2.5 °	

		GISS-E2-R-CC	2 °×2.5 °	
MOHC	Met Office Hadley Centre	HadGEM2-CC	1.25 °×1.875 °	(21)
		HadGEM2-ES	1.25 °×1.875 °	
INM	Institute for Numerical Mathematics	INMCM4	1.5 °×2 °	(22)
IPSL	Institute Pierre-Simon Laplace	IPSL-CM5A-LR	1.89 °×3.75 °	(23, 24)
		IPSL-CM5A-MR	1.89 °×3.75 °	
		IPSL-CM5B-LR	1.89 °×3.75 °	
MIROC	National Institute for Environmental Studies, The University of Tokyo	MIROC-ESM	2.8125 °×2.8125 °	(25)
		MIROC-ESM-CHEM	2.8125 °×2.8125 °	

References

1. A. Ahlström, M.R. Raupach, G. Schurgers, *et al.*, The dominant role of semi-arid ecosystems in the trend and variability of the land CO_2 sink. *Science*, **348**, 895-899 (2015).
2. V. Haverd *et al.*, A new version of the CABLE land surface model (Subversion revision r4601) incorporating land use and land cover change, woody vegetation demography, and a novel optimisation-based approach to plant coordination of photosynthesis. *Geoscientific Model Development* **11**, 2995-3026 (2018).
3. J. Melton, V. Arora, Competition between plant functional types in the Canadian Terrestrial Ecosystem Model (CTEM) v. 2.0. *Geoscientific Model Development* **9**, 323-361 (2016).
4. D. M. Lawrence *et al.*, The Community Land Model version 5: Description of new features, benchmarking, and impact of forcing uncertainty. *Journal of Advances in Modeling Earth Systems* **11**, 4245-4287 (2019).
5. H. Tian *et al.*, Anthropogenic and climatic influences on carbon fluxes from eastern North America to the Atlantic Ocean: A process‐based modeling study. *Journal of Geophysical Research: Biogeosciences* **120**, 757-772 (2015).
6. P. Meiyappan, A. K. Jain, J. I. House, Increased influence of nitrogen limitation on CO2 emissions from future land use and land use change. *Global Biogeochemical Cycles* **29**, 1524-1548 (2015).
7. B. Decharme *et al.*, Recent Changes in the ISBA-CTRIP Land Surface System for Use in the CNRM-CM6 Climate Model and in Global Off-Line Hydrological Applications. *Journal of Advances in Modeling Earth Systems* **11**, 1207-1252 (2019).
8. T. Mauritsen *et al.*, Developments in the MPI-M Earth System Model version 1.2 (MPI-ESM1.2) and Its Response to Increasing CO2. *Journal of Advances in Modeling Earth Systems* **11**, 998-1038 (2019).
9. A. A. Sellar *et al.*, UKESM1: Description and Evaluation of the U.K. Earth System Model. *Journal of Advances in Modeling Earth Systems* **11**, 4513-4558 (2019).
10. S. Lienert, F. Joos, A Bayesian ensemble data assimilation to constrain model parameters and land-use carbon emissions. *Biogeosciences* **15**, 2909-2930 (2018).
11. D. S. Goll *et al.*, Carbon–nitrogen interactions in idealized simulations with JSBACH (version 3.10). *Geosci. Model Dev.* **10**, 2009-2030 (2017).
12. A. P. Walker *et al.*, The impact of alternative trait-scaling hypotheses for the maximum photosynthetic carboxylation rate (Vcmax) on global gross primary production. *New Phytologist* **215**, 1370-1386 (2017).
13. E. Kato, T. Kinoshita, A. Ito, M. Kawamiya, Y. Yamagata, Evaluation of spatially explicit emission scenario of land-use change and biomass burning using a process-based biogeochemical model. *Journal of Land Use Science* **8**,

104-122 (2013).
14. T. Wu *et al.*, An overview of BCC climate system model development and application for climate change studies. *Journal of Meteorological Research* **28**, 34-56 (2014).
15. D. Ji *et al.*, Description and basic evaluation of Beijing Normal University Earth system model (BNU-ESM) version 1. *Geoscientific Model Development* **7**, 2039-2064 (2014).
16. P. Chylek, J. Li, M. Dubey, M. Wang, G. Lesins, Observed and model simulated 20th century Arctic temperature variability: Canadian earth system model CanESM2. *Atmospheric Chemistry and Physics Discussions* **11**, 22893-22907 (2011).
17. P. R. Gent *et al.*, The community climate system model version 4. *Journal of Climate* **24**, 4973-4991 (2011).
18. J. P. Dunne *et al.*, GFDL's ESM2 global coupled climate–carbon earth system models. Part I: Physical formulation and baseline simulation characteristics. *Journal of Climate* **25**, 6646-6665 (2012).
19. J. P. Dunne *et al.*, GFDL's ESM2 global coupled climate–carbon earth system models. Part II: carbon system formulation and baseline simulation characteristics. *Journal of Climate* **26**, 2247-2267 (2013).
20. G. A. Schmidt *et al.*, Present-day atmospheric simulations using GISS ModelE: Comparison to in situ, satellite, and reanalysis data. *Journal of Climate* **19**, 153-192 (2006).
21. W. Collins *et al.*, Development and evaluation of an Earth-System model–HadGEM2. *Geoscientific Model Development* **4**, 1051-1075 (2011).
22. E. Volodin, N. Dianskii, A. Gusev, Simulating present-day climate with the INMCM4. 0 coupled model of the atmospheric and oceanic general circulations. *Izvestiya, Atmospheric and Oceanic Physics* **46**, 414-431 (2010).
23. F. Hourdin *et al.*, Impact of the LMDZ atmospheric grid configuration on the climate and sensitivity of the IPSL-CM5A coupled model. *Climate Dynamics* **40**, 2167-2192 (2013).
24. F. Hourdin *et al.*, LMDZ5B: the atmospheric component of the IPSL climate model with revisited parameterizations for clouds and convection. *Climate Dynamics* **40**, 2193-2222 (2013).
25. S. Watanabe *et al.*, MIROC-ESM: model description and basic results of CMIP5-20c3m experiments. *Geoscientific Model Development Discussions* **4**, 1063-1128 (2011).
